# Observation of the effect of gravity on the motion of antimatter

**DOI:** 10.1038/s41586-023-06527-1

**Published:** 2023-09-27

**Authors:** E. K. Anderson, C. J. Baker, W. Bertsche, N. M. Bhatt, G. Bonomi, A. Capra, I. Carli, C. L. Cesar, M. Charlton, A. Christensen, R. Collister, A. Cridland Mathad, D. Duque Quiceno, S. Eriksson, A. Evans, N. Evetts, S. Fabbri, J. Fajans, A. Ferwerda, T. Friesen, M. C. Fujiwara, D. R. Gill, L. M. Golino, M. B. Gomes Gonçalves, P. Grandemange, P. Granum, J. S. Hangst, M. E. Hayden, D. Hodgkinson, E. D. Hunter, C. A. Isaac, A. J. U. Jimenez, M. A. Johnson, J. M. Jones, S. A. Jones, S. Jonsell, A. Khramov, N. Madsen, L. Martin, N. Massacret, D. Maxwell, J. T. K. McKenna, S. Menary, T. Momose, M. Mostamand, P. S. Mullan, J. Nauta, K. Olchanski, A. N. Oliveira, J. Peszka, A. Powell, C. Ø. Rasmussen, F. Robicheaux, R. L. Sacramento, M. Sameed, E. Sarid, J. Schoonwater, D. M. Silveira, J. Singh, G. Smith, C. So, S. Stracka, G. Stutter, T. D. Tharp, K. A. Thompson, R. I. Thompson, E. Thorpe-Woods, C. Torkzaban, M. Urioni, P. Woosaree, J. S. Wurtele

**Affiliations:** 1 Department of Physics and Astronomy, Aarhus University, Aarhus, Denmark; 2 Department of Physics, Faculty of Science and Engineering, Swansea University, Swansea, UK; 3 School of Physics and Astronomy, University of Manchester, Manchester, UK; 4 Cockcroft Institute, Sci-Tech Daresbury, Warrington, UK; 5 University of Brescia, Brescia and INFN Pavia, Pavia, Italy; 6 TRIUMF, Vancouver, British Columbia Canada; 7 Instituto de Fisica, Universidade Federal do Rio de Janeiro, Rio de Janeiro, Brazil; 8 Department of Physics, University of California at Berkeley, Berkeley, CA USA; 9 Department of Physics and Astronomy, University of British Columbia, Vancouver, British Columbia Canada; 10 Accelerator and Technology Sector, CERN, Geneva, Switzerland; 11 Department of Physics and Astronomy, York University, Toronto, Ontario Canada; 12 Department of Physics and Astronomy, University of Calgary, Calgary, Alberta Canada; 13 Department of Physics, Simon Fraser University, Burnaby, British Columbia Canada; 14 Van Swinderen Institute for Particle Physics and Gravity, University of Groningen, Groningen, The Netherlands; 15 Department of Physics, Stockholm University, Stockholm, Sweden; 16 Department of Physics, British Columbia Institute of Technology, Burnaby, British Columbia Canada; 17 Department of Chemistry, University of British Columbia, Vancouver, British Columbia Canada; 18 Institute for Particle Physics and Astrophysics, ETH, Zurich, Switzerland; 19 Experimental Physics Department, CERN, Geneva, Switzerland; 20 Department of Physics and Astronomy, Purdue University, West Lafayette, IN USA; 21 Accelerator Systems Department, CERN, Geneva, Switzerland; 22 Soreq NRC, Yavne, Israel; 23 Department of Physics, Ben Gurion University, Beer Sheva, Israel; 24 INFN Pisa, Pisa, Italy; 25 School of Mathematical and Physical Sciences, University of Sussex, Brighton, UK; 26 Physics Department, Marquette University, Milwaukee, WI USA

**Keywords:** Experimental particle physics, Exotic atoms and molecules

## Abstract

Einstein’s general theory of relativity from 1915^1^ remains the most successful description of gravitation. From the 1919 solar eclipse^2^ to the observation of gravitational waves^3^, the theory has passed many crucial experimental tests. However, the evolving concepts of dark matter and dark energy illustrate that there is much to be learned about the gravitating content of the universe. Singularities in the general theory of relativity and the lack of a quantum theory of gravity suggest that our picture is incomplete. It is thus prudent to explore gravity in exotic physical systems. Antimatter was unknown to Einstein in 1915. Dirac’s theory^4^ appeared in 1928; the positron was observed^5^ in 1932. There has since been much speculation about gravity and antimatter. The theoretical consensus is that any laboratory mass must be attracted^6^ by the Earth, although some authors have considered the cosmological consequences if antimatter should be repelled by matter^7–10^. In the general theory of relativity, the weak equivalence principle (WEP) requires that all masses react identically to gravity, independent of their internal structure. Here we show that antihydrogen atoms, released from magnetic confinement in the ALPHA-g apparatus, behave in a way consistent with gravitational attraction to the Earth. Repulsive ‘antigravity’ is ruled out in this case. This experiment paves the way for precision studies of the magnitude of the gravitational acceleration between anti-atoms and the Earth to test the WEP.

## Abstract (web summary)

Magnetically confined neutral antihydrogen atoms released in a gravity field were found to fall towards Earth like ordinary matter, in accordance with Einstein’s general theory of relativity.

## Main

The weak equivalence principle (WEP) has recently been tested for matter in Earth’s orbit^11^ with a precision of order 10^−15^. Antimatter has hitherto resisted direct ballistic tests of the WEP due to the lack of a stable, electrically neutral, test particle. Electromagnetic forces on charged antiparticles make direct measurements in the Earth’s gravitational field extremely challenging^12^. The gravitational force on a proton at the Earth’s surface is equivalent to that from an electric field of about 10^−7^ V m^−1^. The situation with magnetic fields is even more dire: a cryogenic antiproton^13^ at 10 K would experience gravity-level forces in a magnetic field of order 10^−10 ^T. Controlling stray fields to this level to unmask gravity is daunting. Experiments have, however, shown that confined, oscillating, charged antimatter particles behave as expected when considered as clocks^14–16^ in a gravitational field. The abilities to produce^17^ and confine^18^ antihydrogen now allow us to employ stable, neutral anti-atoms in dynamic experiments where gravity should play a role. Early considerations^19,20^ and a more recent proof-of-principle experiment^21^ in 2013 illustrated this potential. We describe here the initial results of a purpose-built experiment designed to observe the direction and the magnitude of the gravitational force on neutral antimatter.

## Antihydrogen and ALPHA-g

Trapping and accumulation^22^ of antihydrogen are now routine, with up to several thousand atoms having been simultaneously stored in the ALPHA-2 device^23^. To date, all of the measurements of the properties of antihydrogen^24–29^ have been performed in ALPHA magnetic traps. In 2018, the ALPHA-g machine—a vertically oriented antihydrogen trap designed to study gravitation—was constructed. The experimental strategy is conceptually simple: trap and accumulate atoms of antihydrogen; slowly release them by opening the top and bottom barrier potentials of the vertical trap; and try to discern any influence of gravity on their motion when they escape and annihilate on the material walls of the apparatus. The trapped anti-atoms are not created at rest but have a distribution of kinetic energies consistent with the trap depth of about 0.5 K (we employ temperature-equivalent energy units). Gravity is expected to be manifested as a difference in the number of annihilation events from anti-atoms escaping via the top or the bottom of the trap.

**Fig. 1:**
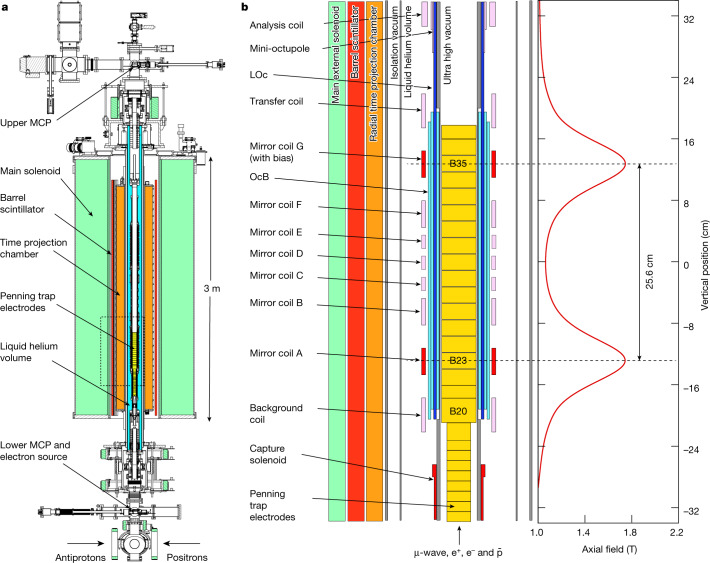
ALPHA-g apparatus. **a**, Cross section of the ALPHA-g apparatus. The full device comprises three antihydrogen trapping regions; only the bottom one is employed here. The MCP detectors are used to image charged particles (e^−^, e^+^, $\bar{{\rm{p}}}$) extracted from the Penning traps for diagnostic purposes. **b**, Expanded view of the bottom antihydrogen trap (the dashed rectangle in **a**) illustrating the Penning trap for antihydrogen production and the superconducting coils that form the neutral atom trap. The on-axis, axial field profile at full current is shown on the right. Note that the rTPC, the barrel scintillator and the main solenoid are not drawn to scale here; see Fig. 1a for a scaled image. The mirror coils B–F, the analysis coil, the mini-octupole, the transfer coil and the background coil are not utilized here. The capture solenoid is used for charged particle transfer and manipulations and is de-energized for gravity measurements. The LOc coils (dark blue in the figure) extend past the trapping region used here and constitute part of two additional antihydrogen traps intended for future use.

The experimental layout is shown in Fig. 1. Antiprotons from the CERN Antiproton Decelerator^30^ and the ELENA (Extra Low ENergy Antiproton)^31^ ring are first caught in a separate, high voltage Penning trap in a 3 T solenoid magnet (not shown). ELENA typically delivers 7.5 × 10^6^ antiprotons at 100 keV every 120 s. About 5 × 10^5^ of these are dynamically captured. After being cooled by co-trapped electrons, antiprotons are injected into ALPHA-g and dynamically re-trapped. A superconducting solenoid provides the background field of 1 T for confining the charged particles. Positrons from a Surko-type accumulator^32^ are also injected into ALPHA-g and re-trapped; there are typically 3 × 10^6^ available for each mixing cycle with antiprotons. The beamline for guiding the bunches of positrons and antiprotons into ALPHA-g is described elsewhere^33^. Following manipulations to control their size and density^34^, the positron plasmas are mixed with antiproton plasmas in a region (electrodes B23 to B35 in Fig. 1) situated within the superconducting antihydrogen trap. The anti-atom trap comprises octupole magnets for transverse confinement and two solenoidal ‘mirror coils’ (A and G in Fig. 1) for axial (vertical) confinement. Antihydrogen atoms produced with sufficiently low kinetic energy can be trapped due to the –**μ**•**B** interaction of their magnetic moments with the external fields. For the field strengths in ALPHA-g, the anti-atoms are spin-polarized, and the scalar magnitude of the magnetic field determines the trapping potential. The entire production and trapping region is cooled to near 4 K by the liquid helium bath for the trap magnets. ALPHA-g currently traps a few antihydrogen atoms per mixing cycle, but antihydrogen atoms can be accumulated^22^ over many cycles from ELENA. We refer to this process as ‘stacking’. The atom trapping volume is nominally a vertical cylinder of 4.4 cm diameter and 25.6 cm height.

## The effect of gravity

The experimental protocol was to stack antihydrogen atoms, then release them by ramping down the current in the two mirror coils simultaneously over 20 s. The anti-atoms could escape either to the top of the trap (through mirror G) or the bottom (through mirror A) and subsequently annihilate on the walls of the apparatus (Fig. 1). The annihilations and their positions (vertices) could be detected and reconstructed using the ALPHA-g radial time projection chamber (rTPC) detector (Fig. 1 and Methods). A coaxial, barrel-shaped scintillator detector was also used for event selection (Fig. 1 and Methods).

Numerical simulations of atom trajectories (Methods) indicate that if hydrogen atoms were trapped and gradually released from a vertically symmetric trap (that is, the on-axis magnetic field maxima are equal; *B*_A_ = *B*_G_) under ALPHA-g conditions, about 80% of them would exit through the bottom, the asymmetry being due to the downward force of gravity. The goal of the current experiment was to test this behaviour for antihydrogen. Vertical gradients in the magnetic field magnitude can obviously mimic the effect of gravity. Quantitatively, the local acceleration of gravity *g*, which is about 9.81 m s^−2^, is equivalent to a vertical magnetic field gradient of 1.77 × 10^−3 ^T m^−1^ acting on a hydrogen atom in the ground state. The peaks in the mirror coil axial field strength are separated by 25.6 cm at full current, so a field difference of 4.53 × 10^−4^ T between these points would mimic gravity. This consideration sets the scale for the required degree of magnetic field control for this experiment, but it also allows us to refine the simple, symmetric release procedure to more systematically probe gravity. In particular, it is possible to either counteract or supplement gravity by introducing a differential current to one of the mirror coils.

**Fig. 2:**
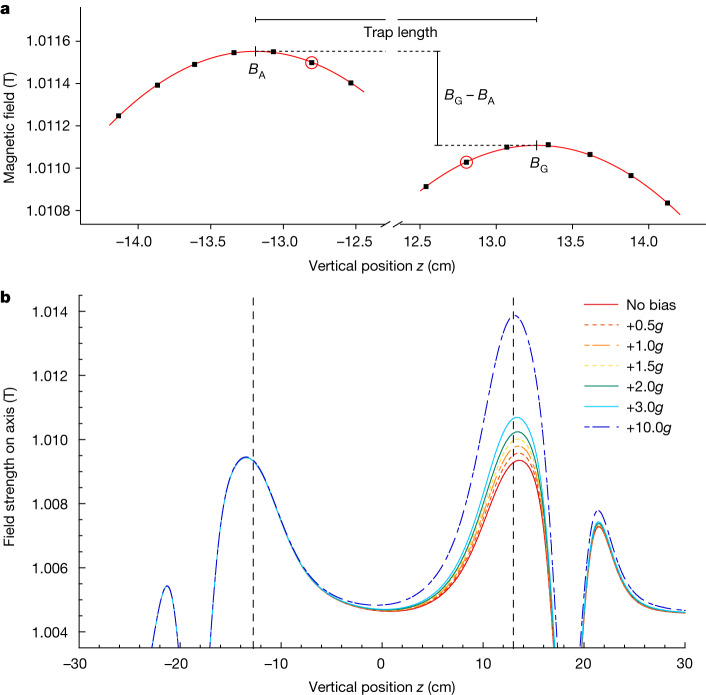
Illustrations of the magnetic bias. **a**, Expanded view of the end-of-ramp mirror coil peak regions for a bias of −1*g* (note the discontinuous abscissa). The square points represent offline ECR measurements carried out to determine the field profile and to find the peak field location. The points with red circles indicate the axial locations at which ECR measurements were made at the beginning and end of the mirror coil ramp-down for each gravity trial. **b**, Calculated on-axis final well shapes (after ramp-down) for the positive bias trials. The features at |*z*| > 20 cm are due to the OcB (Fig. 1) end turn windings. The vertical dashed lines represent the physical axial midpoints of mirrors A and G.

We first consider a simplified, one-dimensional on-axis model. As the mirror fields are ramped down, a particular anti-atom will escape when its axial kinetic energy exceeds the combined gravitational and magnetic potential at the peak axial field position of one of the mirror coils. Thus, one could balance the effect of gravity on matter by imposing a field difference (*B*_G_ − *B*_A_) of about −4.53 × 10^−4 ^T between the mirror field peaks (Fig. 2a). Maintaining this difference during the ramp-down would in principle result in half of the atoms escaping in each direction. Note that this incremental field is very small compared to the size of the initial peak end field, which is about 1.74 T. The mirror coils A and G were connected in series, and a bipolar current supply connected only to mirror G could provide a field increment or decrement (Methods). We emphasize that a magnetic gradient is not applied uniformly over the length of the trap. The local field geometry in the region of each mirror coil determines which particles can escape axially.

## The release experiment

In anticipation of future precision experiments, the octupole fields in ALPHA-g can be generated by three distinct coils. Two of these, which we designate long octupole (LOc) and bottom octupole (OcB), are employed here (Fig. 1). The OcB magnet (made up of six wound current layers) spans the axial trapping region employed in the current experiment. The LOc magnet comprises two layers of windings and extends over 1.5 m of the apparatus, covering two additional antihydrogen trapping regions not utilized here. For trapping and stacking, both octupole magnets are energized to about 830 A. At the completion of stacking, the LOc magnet is ramped down in 1 s, thereby eliminating the transverse confinement field above mirror G (Fig. 1). This step releases some of the more transversely energetic atoms – about half of the stacked sample. By counting the resulting annihilations, we obtain an indication of the total number of atoms that have been stacked.

The actual experiment involved many trials of antihydrogen accumulation and release for various magnetic ‘bias’ levels. We define the imposed bias as:

$$\frac{{{\rm{\mu }}}_{{\rm{B}}}({B}_{{\rm{G}}}-{B}_{{\rm{A}}})}{{m}_{{\rm{H}}}({z}_{{\rm{G}}}-{z}_{{\rm{A}}})}$$

where ${\mu }_{{\rm{B}}}$ is the Bohr magneton, $({B}_{{\rm{G}}}-{B}_{{\rm{A}}})$ is the difference between the on-axis field maxima under the two mirror coils, ${m}_{{\rm{H}}}$ is the hydrogen gravitational mass and $({z}_{{\rm{G}}}-{z}_{{\rm{A}}})$ is the height difference between the positions of the on-axis field maxima. It is convenient to express the bias relative to *g*. Thus, in the one-dimensional model, a magnetic bias of −1*g* would effectively balance the downwards gravitational force for hydrogen. Having assumed no a priori direction or magnitude for the gravitational force on antihydrogen, we investigated nominal bias values of ±3*g*, ±2*g*, ±1.5*g*, ±1*g*, ±0.5*g* and 0*g*. Figure 2b illustrates the positive bias fields (*B*_G_ > *B*_A_), which would encourage antihydrogen atoms to exit at the bottom.

We typically accumulated anti-atoms for 50 stacks in roughly four hours, resulting in about 100 atoms trapped. For each trial, after the conclusion of stacking and the LOc ramp-down, the on-axis field magnitude at one axial location under each mirror coil (Fig. 2a) was measured using the technique of electron cyclotron resonance (ECR)^35^ (Methods). The ECR measurement was made at approximately 130 s after the LOc ramp-down. The mirror coil current ramp-downs happened next and were linear over 20 s. The smaller of the two mirror fields was not ramped all the way down to the level of the bottom of the confinement well but stopped at about 5 × 10^−3^ T above this level. This was to ensure that the released atoms possessed enough energy to overcome the small axial field bumps that arise from the end windings of the OcB magnet (Fig. 2b). At approximately 96 s after the mirror ramp-down, the ECR measurements were repeated to characterize the final axial well (Methods).

**Fig. 3:**
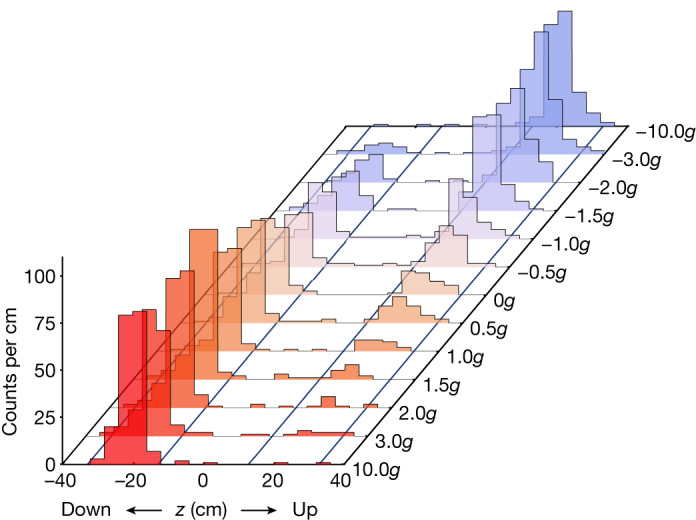
Escape histograms. The raw event *z-*distributions are displayed as histograms for each of the bias values, including the ±10*g* calibration runs. These are uncorrected for background or detector relative efficiency. The time window represented here is 10 s to 20 s of the magnet ramp-down. The *z*-cut regions are indicated by the solid, diagonal lines. Explicitly, the acceptance regions in *z* are [−32.8, −12.8] and [12.8, 32.8] cm for the ‘down’ and ‘up’ regions, respectively.

**Fig. 4:**
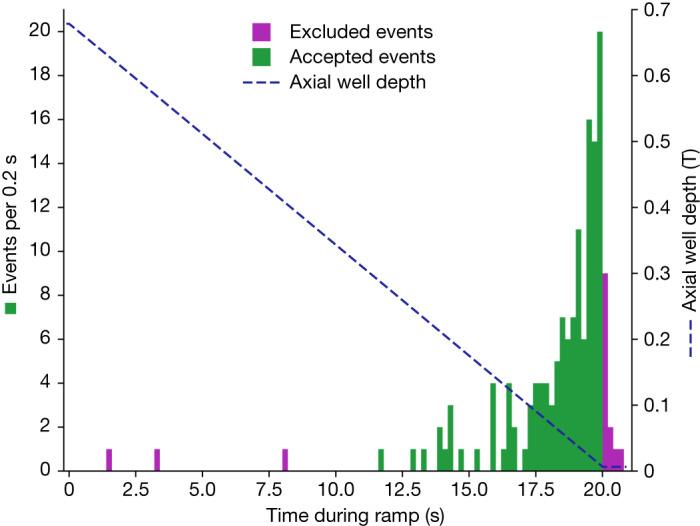
Time structure of the annihilation events from escaped antihydrogen. The number of detected events (left ordinate) is plotted as a function of time as the magnets are ramped down. This figure represents the sum of the seven trials having bias 0*g*. The dashed line (right ordinate) illustrates the calculated axial well depth during the magnet ramp-down. The excluded events fail the time cut.

Various bias values were interleaved during the data-taking period, which lasted about 30 days. We emphasize that the integer or half-integer bias values identified above are just labels for the trials and refer to the programmed on-axis field maxima; neither is the bias perfectly constant during the ramp-down, nor does the one-dimensional model completely characterize the three-dimensional experiment. Trials for a given bias were repeated six or seven times, depending on the total number of events detected. The raw results (no background subtraction or detector efficiency correction) are presented as axial annihilation distributions in Fig. 3. For further analysis, we exclude events whose *z*-position lies between the physical mirror centres, or more than 0.2 m outside the physical mirror centres, as indicated in Fig. 3. This ‘*z*-cut’ was chosen by conducting a separate set of experiments in which we attempted to release trapped antihydrogen atoms to only the top or the bottom of the trap by applying a bias of −10*g* or +10*g*, respectively. The ±10*g* trials also help to determine the relative efficiency of the rTPC detector for the up and down escape regions (Methods). The efficiency determination uses the number of atoms detected in the LOc ramp-down as a normalization. The plotted event distributions were also subject to a ‘time cut’: events are accepted from 10 to 20 s of the ramp-down, as we found that the number of atoms emerging before 10 s is negligible (Fig. 4).

The essential cumulative result for each bias can be represented by two numbers, *N*_up_ and *N*_dn_: the number of particles escaping upwards or downwards. These are listed in Table 1. The techniques used to maximize the signal and suppress the background are described in Methods. The background rates are listed in the Table 1 notes.

**Table 1:** Results of the release trials

Nominal bias (*g*)	Number of trials	*N*_up_ (events)	*N*_dn_ (events)	Events during LOc ramp-down
−3.0	7	151.7	16.5	199.2
−2.0	7	128.7	33.5	168.2
−1.5	6	128.9	57.7	192.0
−1.0	7	69.7	62.5	183.2
−0.5	7	55.7	67.5	201.2
0	7	36.7	94.5	144.2
0.5	7	36.7	124.5	177.2
1.0	7	17.7	119.5	185.2
1.5	6	13.9	180.7	234.0
2.0	7	6.7	163.5	228.2
3.0	7	7.7	147.5	199.2
−10.0	6	142.9	0.7	169.0
10.0	6	−0.1	185.7	213.0

**Fig. 5:**
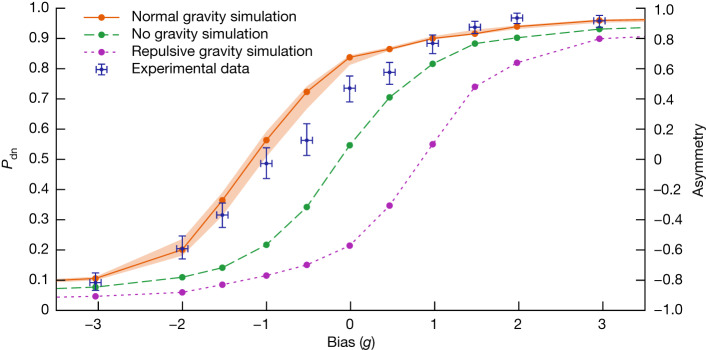
Escape curve and simulations. The derived *P*_dn_ values are plotted versus bias for the experimental data and for simulations of the experiment for three values of the gravitational acceleration ${a}_{\bar{g}}$: 1*g* (normal gravity, orange), 0*g* (no gravity, green) and −1*g* (repulsive gravity, violet). See the text for the definitions of the uncertainties. The right ordinate is the down-up asymmetry *A* *=* 2P_dn _− 1. The confidence intervals on the no- and repulsive gravity simulations are comparable to those for the normal gravity simulation and have been omitted for clarity.

The number of events for anti-atoms escaping either up or down is tabulated for each bias series. These events occur in the time window 10–20 s during the ramp-down and lie within the *z*-regions illustrated in Fig. 3. Also shown is the number of events due to antihydrogen atoms that escape when the long octupole magnet is ramped down. All values are corrected for the expected cosmic ray background. Counting uncertainties are not listed but are used in the global determination of *P*_dn_ in Fig. 5. The background per trial was 0.18 ± 0.01 events in the top region and 0.21 ± 0.01 events in the bottom region. The background per trial for the LOc ramp-down window (duration 13.1 s) was 0.83 ± 0.02 events. The ±10*g* entries are for the calibration trials (see text).

## The escape curve

In Fig. 5 we plot the probability for an antihydrogen atom to escape downwards (*P*_dn_) as a function of the applied bias. The probabilities and their credible intervals were obtained from the raw event counts by using standard statistical techniques (Methods). The biases plotted here are derived values, as the magnetic field difference (on axis) between the upper and lower barriers remains only approximately constant as the current is decreased. This is due to small asymmetries in the background field, the construction of the mirror coils and the ramp-induced persistent currents in the superconductors (Methods). We also observe that these currents decay after the end of the ramp (Extended Data Fig. 6), affecting the final-well ECR measurements. To account for these effects, we use a measurement-based magnetic field model (Methods) to calculate the bias during the ramp. We can then assign to each annihilation event the calculated bias for the time at which that particular anti-atom escaped the trap (Fig. 4 and Extended Data Fig. 8). Finally, we average the biases for all of the events that pass our selection criteria (or ‘cuts’) to arrive at the plotted bias value for the collection of trials sharing the same magnetic field configuration. The uncertainties in the bias determination are of order 0.1*g* and are described in detail in Methods.

Qualitatively, the experimental data in Fig. 5 exhibit the behaviour characteristic of gravitational attraction between antihydrogen and the Earth. At a bias of about +3*g*(−3*g*) the anti-atoms exit predominantly at the bottom(top) of the trap, as the magnetic imbalance is significantly larger than 1*g*. The fraction exiting through the bottom increases monotonically as the bias increases from −3*g* to +3*g*. The balance point (*P*_dn_ = 0.5) is close to −1*g*, as naively expected from the simplified one-dimensional argument presented above.

To gain more quantitative insight into the results (and originally to inform the design of the experiment) we rely on extensive numerical simulations (Methods) of the trajectories of antihydrogen atoms trapped and then released. The numerical model features a three-dimensional magnetic field map based on both the as-built superconducting magnet wire model and the measured fields from ECR or a magnetron frequency measurement technique (Methods). The actual currents measured during the experimental sequence are used for the simulation. This is the same magnetic field model used to derive the plotted biases above, so the simulation describes a three-dimensional system that is consistent with our best experimental measurements—both static and dynamic—of on-axis field strengths. The ECR measurements taken during the trials have been supplemented by extensive offline studies using both ECR and the magnetron method (Methods). The simulated release results are plotted with the data in Fig. 5, both for attractive (normal) gravity and, by way of comparison, for ‘no’ gravity and for ‘repulsive’ gravity.

**Extended Data Fig. 1:**
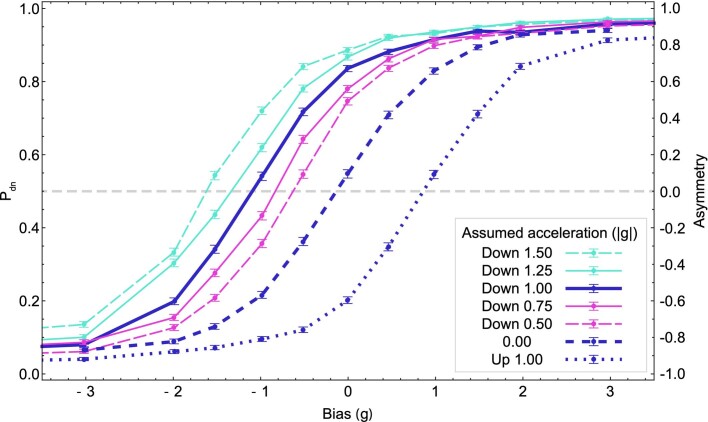
Simulated escape curves for various values of ${a}_{\bar{g}}$. We illustrate the escape curves resulting from assuming several different values of the gravitational acceleration, ${a}_{\bar{g}}$, of antimatter due to the Earth. See the legend for details. The simulations are otherwise identical to that used for the 20 s release experiment and normal gravity (Fig. 5). The solid blue curve represents expectations for normal gravity.

The agreement between the shape of the measured data and that of the simulation is visually compelling. To extract a value for the local acceleration from our dataset, we have compared the data to a set of simulations that presume values for antihydrogen’s gravitational acceleration that differ from 1*g* (Extended Data Fig. 1). Generally speaking, the simulated curves have the same shape and are shifted along the bias axis. From a likelihood analysis (Methods) on the experimental data, we find that the local gravitational acceleration of antihydrogen is directed towards the Earth and has magnitude ${a}_{\bar{{\rm{g}}}}$ = (0.75 ± 0.13 (statistical + systematic) ± 0.16 (simulation))*g*, where *g* = 9.81 m s^−2^. Within the stated errors, this value is consistent with a downward gravitational acceleration of 1*g* for antihydrogen.

## Classification of uncertainties

Broadly speaking, we characterize three different types of uncertainty. The uncertainties regarding magnetic field measurement and modelling affect the derived bias values and are listed in Table 2 and described in Methods. These are reflected in the horizontal error bars on the bias values in Fig. 5. Statistical and systematic uncertainties regarding event detection, such as counting statistics, backgrounds and detector efficiencies, are listed in Table 3. These are manifested as vertical error bars in the *P*_dn_ values in Fig. 5. Finally, an estimated uncertainty band (orange band in Fig. 5) is associated with the simulation. This includes the potential impact of various unmeasured quantities, such as magnet winding misalignments, off-axis persistent magnetic fields, and uncertainty in the energy distributions (longitudinal and transverse) of the trapped antihydrogen atoms. All of the above are used to extract the uncertainties in the quoted value of ${a}_{\bar{{\rm{g}}}}$. Our goal here is not to make a precision determination of the magnitude of ${a}_{\bar{{\rm{g}}}}$, but to identify the statistical sensitivities and systematic effects that will be important for future measurements.

**Table 2:** Uncertainties in the bias determination

Uncertainty	Magnitude (*g*)
ECR spectrum width	0.07
Repeatability of $({B}_{{\rm{G}}}-{B}_{{\rm{A}}})$	0.014
Peak field size and *z*-location fit	0.009
Field decay asymmetry (A to G) after ramp	0.02
Bias variation in time	0.02
Field modelling	0.05

Summary of the uncertainties in the derived bias values, expressed in units of the local acceleration of gravity for matter (9.81 m s^−2^). See Methods for definitions and details.

**Table 3:** Uncertainties in the determination of ${a}_{\bar{{\rm{g}}}}$

	Uncertainty	Magnitude (*g*)
Statistical and systematic	Finite data size	0.06
	Calibration of the detector efficiencies in the up and down regions	0.12
	Other minor sources	0.01
Simulation model	Modelling of the magnetic fields (on-axis and off-axis)	0.16
	Antihydrogen initial energy distribution	0.03

Summary of the uncertainties involved in the determination of the gravitational acceleration ${a}_{\bar{{\rm{g}}}}$. The uncertainties are one standard deviation and are expressed in units of the local acceleration of gravity for matter (9.81 m s^−2^). See Methods for the details.

**Extended Data Fig. 2:**
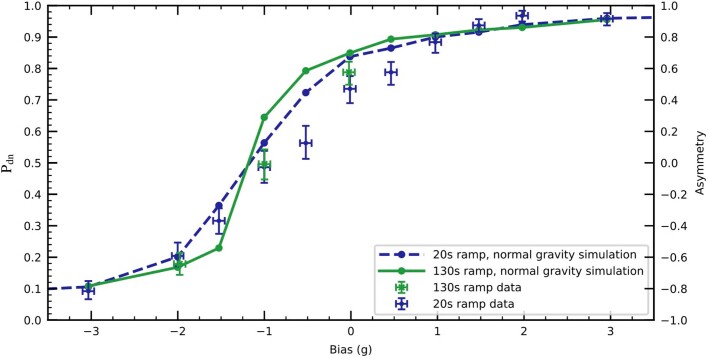
Escape curve for 130 s ramp-down. The *P*_dn_ values are plotted versus bias for the three trial sets having biases 0 *g*, −1 *g*, and −2 *g*. These biases were chosen after the 20 s ramp results had been examined. Apart from the slower ramp, the experimental and analysis procedures were identical to those for the 20 s protocol. The 20 s data and simulations for both ramp times are also shown for comparison. Note that the simulated escape curve for the 130 s ramp has a steeper transition region than for the 20 s ramp, and the balance point (*P*_dn_ = 0.5) is not at a bias of precisely −1 *g*, as described in the text.

As a cross check, we conducted trials in which we used a 130 s ramp-down time, for biases of 0*g*, −1*g* and −2*g*. Within the calculated uncertainties, the results were consistent with the 20 s data and with the appropriate simulation (Extended Data Fig. 2).

**Extended Data Fig. 3:**
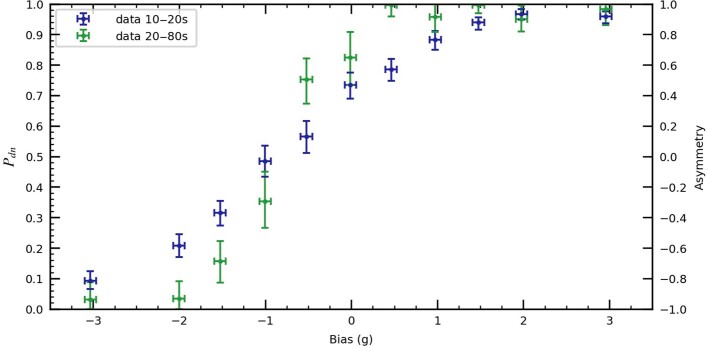
Escape curve for atoms escaping after 20 s. The escape curve (green points) for the time period (20–80 s) after the mirrors A and G have stopped ramping down and are held at constant current while the ECR measurement is prepared. The main (10–20 s) data set (blue points) is shown for comparison. Note that the bias values and their uncertainties for the green points are assumed to be the same as for the blue points. This assumption should be valid within the uncertainties. The vertical error bars are obtained by following the same procedure described in the text. The blue (green) curve is based on 1,722 (621) total events as defined in Table 1.

We also observe that some atoms are released after the end of the 20 s ramp (Fig. 4 and Extended Data Fig. 3). This is potentially due to long-time-scale mixing^36^ between the transverse and longitudinal motions of the atoms, but this has not yet been investigated in detail. The gravitational behaviour of these atoms appears to be consistent with the 20 s ramp-down sample (Extended Data Fig. 3), but the detailed systematic measurements to confirm this have not yet been performed.

## Possible complicating effects

We have considered other effects that could mimic a gravitational force or add significant uncertainty, and we can rule them out due to their negligible magnitudes. We have earlier determined an experimental limit for the antihydrogen charge^37^ to be less than about 10^−28 ^C. Thus, a 1 V potential change would have the same effect as a 10^−5 ^T change in magnetic field. The trap electrodes are maintained at their common ground to within ±10 mV after stacking is completed, so even the extremely unlikely presence of the maximal non-zero charge on antihydrogen would play no role here. Concerning the size of the magnetic dipole moment of antihydrogen, we earlier measured the microwave transition^25^ within the hyperfine-split ground state at approximately 1 T with an absolute accuracy corresponding to 0.3 mT. Since the positron magnetic dipole moment mainly determines the transition frequency, this corresponds to an uncertainty of the magnetic dipole moment of less than 1 part per thousand in antihydrogen, leading to a negligible contribution to the error budget here.

The measured masses and charges of the positron and antiproton^38^ can, in the absence of new physics, be used to constrain the polarizability of an antihydrogen atom in the ground state to approximately that of the hydrogen ground state^39^: 7.4 × 10^−41 ^C^2 ^(J m)^−1^. Thus, a change in electric field of 100 V m^−1^ would have an effect equivalent to a change in magnetic field of less than 10^−13 ^T. Finally, antihydrogen atoms may change their velocity due to collisions with background gas during the ramp-down. From the measured antiproton storage lifetime of 4,000 s in the trap, we estimate the density of background gas to be approximately 2 × 10^5 ^cm^−3^. Using this value together with the calculated cross sections^40^, the probability for a collision during the 20 s (130 s) ramp-down is less than 0.5% (3%).

## Conclusion

We have searched for evidence of the effect of gravity on the motion of particles of neutral antimatter. The best fit to our measurements yields a value of (0.75 ± 0.13 (statistical + systematic) ± 0.16 (simulation)) *g* for the local acceleration of antimatter towards the Earth. We conclude that the dynamic behaviour of antihydrogen atoms is consistent with the existence of an attractive gravitational force between these atoms and the Earth. From the asymptotic form of the distribution of the likelihood ratio as a function of the presumed acceleration, we estimate a probability of 2.9 × 10^−4^ that a result, at least as extreme as that observed here, could occur under the assumption that gravity does not act on antihydrogen. The probability that our data are consistent with the repulsive gravity simulation is so small as to be quantitatively meaningless (less than 10^−15^). Consequently, we can rule out the existence of repulsive gravity of magnitude 1*g* between the Earth and antimatter. The results are thus far in conformity with the predictions of General Relativity. Our results do not support cosmological models relying on repulsive matter–antimatter gravitation.

## Future perspectives

This experiment marks the beginning of detailed, direct inquiries into the gravitational nature of antimatter. Having determined the sign and approximate magnitude of the acceleration, our next challenge is to extend the method to measure the magnitude as precisely as possible, to provide a more stringent test of the WEP. Colder atoms will obviously allow for more sensitive measurements, and our simulations indicate that using colder antihydrogen atoms will in general steepen the transition region of the escape curve and allow for higher precision. Our recent demonstration of laser cooling of trapped antihydrogen^23^ is a promising development in this direction. Additionally, our future measurements will incorporate adiabatic expansion cooling of trapped antihydrogen^41^. In addition to future measurements in ALPHA-g, alternative approaches are being pursued by the GBAR^42^ and AEgIS^43^ collaborations at CERN.

The dependence on simulations is not a concern at the current level of precision, but supplementary experiments to benchmark and refine the simulations will form a large part of the future measurement programme. Our experimental technique is ultimately limited by the precision of the control and measurement of the magnetic fields in the atom trap and its surroundings. Offline magnetometry using electrons, nuclear magnetic resonance^44^ (NMR) probes, and possibly trapped, laser cooled ions^45^, will lead to refinement of the current method. The central trapping region of ALPHA-g, not yet utilized, is designed to be less susceptible to unprogrammed magnetic fields and to work with colder atoms. Having a cold source of stable antimatter in a vertical trap suggests the possibility of performing fountain-type, gravitational interferometry measurements^46^, promising precisions of order 10^−6^ in the determination of ${a}_{\bar{{\rm{g}}}}$. Formerly the subject of countless thought experiments and indirect inferences, the motion of antimatter in the gravitational field of the Earth finally has a sound and promising experimental foothold

## Methods

### Detection of antihydrogen annihilations

#### The ALPHA-g radial time projection chamber

The rTPC is a three-dimensional (3D) particle tracking detector, designed to reconstruct the antihydrogen annihilation location from the charged π-mesons released in the process^47^. The detector has a cylindrical structure, placed between the ALPHA-g trap and the 1 T solenoid magnet (Fig. 1). The ionization electrons created as a charged π-meson passes through the gas medium (mixture of 70% Ar and 30% CO_2_) drift to the outer walls of the detector, where they are collected, generating a read-out signal. The axial (*z*), azimuthal (*ϕ*) and radial (*r*) position information about the particle trajectories is inferred from the signals induced on the segmented cathode pads (4 mm *z* pitch) and anode wires (4.5 mm, or 1.4° *ϕ* pitch), as well as from the drift time—the time it took the electrons to reach the outer wall (typically on order of microseconds). The 1.8 × 10^5 ^cm^3^ active gas volume is 230 cm tall in *z*, and extends from the inner cathode wall (*r* = 10.9 cm) to the segmented outer cathode wall (*r* = 19.0 cm). The gas volume consists of two regions: a drift region (*r* = 10.9 to 17.4 cm), where the main tracking information is obtained, and a proportional region (*r* = 17.4 to 19.0 cm), where electron multiplication takes place, inducing signals on 256 anode ‘sensing wires’ and on the outer cathode pads. The pads have a 576-fold segmentation in *z* and 32-fold in *ϕ* (11.25°), for a total of 18,432 readout channels. A radial drift electric field (*E*_r_) is applied orthogonal to the axial solenoidal magnetic field (*B*_*z*_), making this a relatively uncommon configuration for a TPC^48,49^. This design choice was driven by factors including (1) the large aspect ratio of the height (approximately 230 cm) to the radial width (approximately 10 cm) of the available space, (2) the influence of the non-uniform magnetic fields (from the internal magnets and the solenoid fringe field) on the charge drift and (3) the capability to operate the detector at a lower or zero *B*_*z*_. Due to this field configuration, an electron that drifts radially outwards due to *E*_*r*_ is also displaced in *ϕ*, when *B*_*z*_ is present (Lorentz displacement). The angular deflection is around 9° for maximal drift length at *B*_*z*_ = 1 T.

***The barrel scintillator.*** The barrel scintillator (BSC) surrounds the rTPC and provides additional information on annihilation events. In this work, it was mainly used to provide information on the event topology, as a part of the cosmic background rejection analysis (see below). The BSC is composed of 64 trapezoidal scintillator bars (Eljen Technology EJ-200) that are 2.6 m long and 2 cm thick. The bars are read out at each end by an array of six silicon photomultipliers of type SensL J-series, each photomultiplier having an active area of 6 mm × 6 mm. The analogue signals from the six SiPMs at each end (top or bottom) of a BSC bar are summed in a front-end card on the detector and sent through 5 m coaxial cables to a digitizer module and a time-to-digital converter for each of the 128 channels.

**Extended Data Fig. 4:**
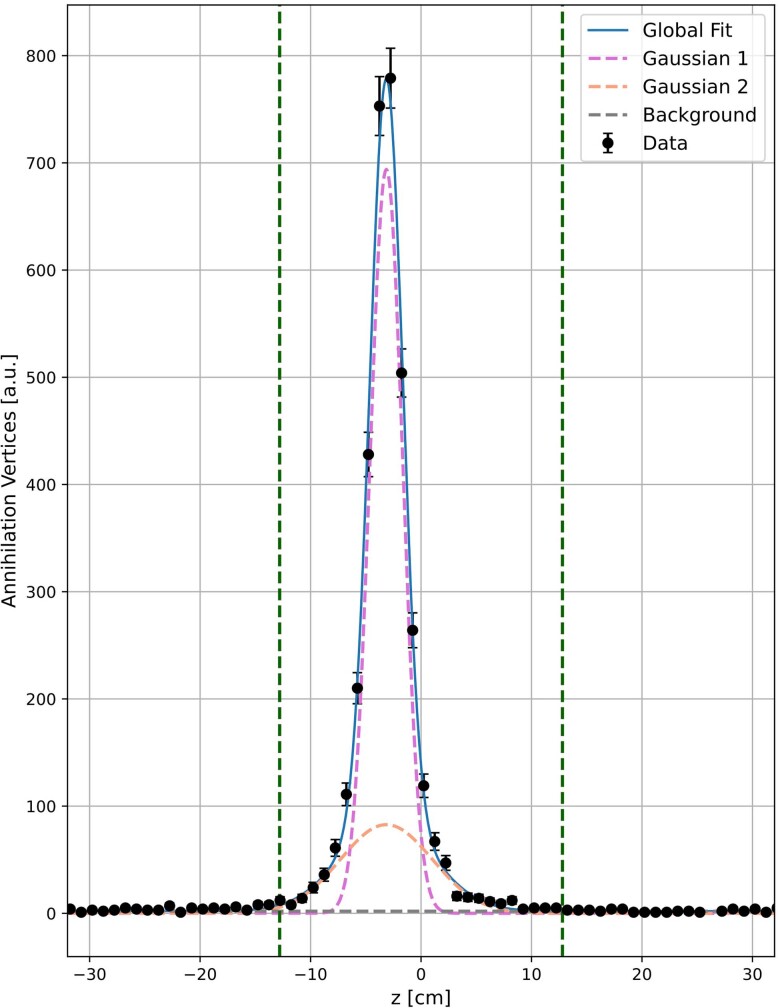
rTPC resolution: a measured *z*-distribution of annihilation vertices from antiprotons held in a Penning trap. An approximately point-like source of events is obtained from antiprotons annihilating on residual gas while being held for 2000 s in a short Penning trap. The reconstructed vertex distribution in *z* (points with error bars) is fitted with two Gaussians and a flat background. The two distributions have standard deviation 1.5 cm (Gaussian 1; ~70% of the counts) and 4.2 cm (Gaussian 2, ~24% of the counts). Both widths are significantly smaller than the distance between mirrors A and G (25.6 cm, magnet centres illustrated by the green vertical lines).

***Reconstruction.*** A charged π-meson typically produces about three ionization clusters per millimetre of track length in the rTPC drift volume. The determination of the 3D position (space point) of the cluster from the detected signals in the pads and the wires requires a model of the charge drift process in the detector gas medium. We use a simulation^50^ based on Garfield++ that accounts for Lorentz displacement in the non-uniform *E*_*r*_ and *B*_*z*_ fields for a given gas condition. In our rTPC configuration, multiple clusters from the same incoming particle often register signals on the same wires or pads. A templated-based deconvolution method was used to infer the space points for these events. Given the set of space points, the particle trajectories are identified using an algorithm that finds the nearest neighbouring point. A least-squares method is employed to find the best fit with a functional form of a helix in three dimensions. Finally, the antihydrogen annihilation position, or vertex, is calculated by finding the point where at least two helices pass closest to each other^47^. Extended Data Fig. 4 shows a distribution of the reconstructed vertices from antiproton annihilations in a Penning trap, indicating a *z* vertex resolution of order 2 cm, which is considerably smaller than the 25.6 cm separation of the mirror coils A and G.

***Machine-learning suppression of the cosmic ray background.*** Cosmic rays are the dominant source of background. The cosmic event rate of around 70 Hz is suppressed by a factor of approximately 350 by offline machine-learning analysis. Twenty selection variables that are sensitive to the topological differences between annihilation and background events are used as inputs to a boosted decision tree classifier^51^. The machine-learning classifier is trained using experimental datasets of signal and background events. The signal sample (371,362 events) was obtained from antihydrogen produced during antiproton and positron mixing near *z* = 0, filtered to only include periods of high event rates to minimize cosmic contamination. The background sample (610,942 events) was collected when there were no antiprotons in the apparatus. None of the machine-learning variables in the training samples showed significant correlation with the vertical axis (*z*). A classifier cut was chosen to optimize the significance for an expected 1 count of signal in a period of 8 seconds. This yields a detection efficiency of 0.65 ± 0.02 annihilations per readout trigger. The background rates depend slightly on *z* and are reported in the Table 1 caption.

***Top and bottom detection efficiencies.*** The gravity measurement requires knowledge of the relative detection efficiencies for antihydrogen released in the defined ‘up’ and ‘down’ regions of the trap. The π-mesons from antihydrogen annihilating in these regions traverse slightly different amounts of material and magnetic fields. As discussed in the main text, the ±10*g* datasets provide a direct calibration of the relative detection efficiencies, because the vast majority of annihilations occurs in only one region. We have performed two other calibration measurements: (1) similar to the above but with a slightly different antihydrogen release sequence and (2) a measurement where only one mirror was ramped down at a time, with the other being held at full field. These measurements gave a consistent relative efficiency, corroborating our ±10*g* measurement.

#### Detector performance and laser calibration

The stability of the detector high voltage, gas flow, temperature, and pressure were closely monitored during the measurement campaign; no trends that would affect the detector efficiency were observed. Regular accumulations of cosmic ray events were taken to monitor detector occupancy, noise levels and background rates. Throughout the campaign, 100% of the rTPC wires, more than 97% of the rTPC pads and more than 99% of the BSC channels were active. Data from faulty or noisy channels were removed from the analysis.

**Extended Data Fig. 5:**
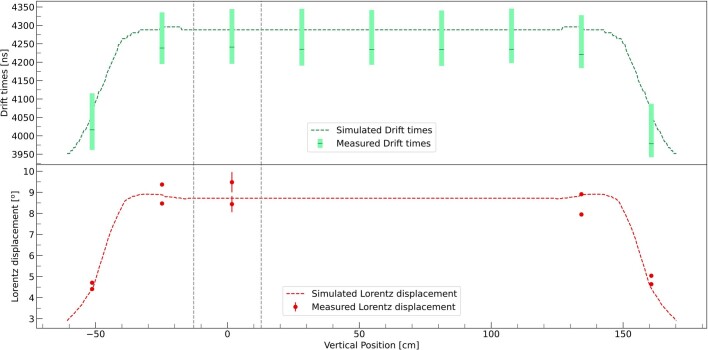
Laser based rTPC calibration and comparison with Garfield++ simulation predictions. **a** The green bars denote the measured drift times of laser-induced photoelectrons released from nine aluminium strips on the inner cathode surface of the rTPC (Methods). The green dashed line is the predicted drift time from the Garfield++ model used in the detector physics analysis. Each green bar denotes the range of drift times measured during the period of the physics measurement campaign, while the dash in each bar denotes the average drift time over those measurements. Vertical dashed lines indicate the axial midpoints of mirrors A and G. **b** The red circles denote measurements, at five *z*-locations, of the Lorentz displacement, i.e., the azimuthal displacement of the radially drifted photoelectrons at the location of the anode wires (*r* = 18.2 cm). The error bars are due to the fit error and are smaller than the plotting symbols except at the vertical position 0 cm. The two points for each *z* are measured at different initial azimuthal positions. The red dashed line denotes the prediction from the same Garfield++ simulation model. Note that vertical scales are magnified for both figures.

A dedicated calibration system was developed to validate the Garfield++ charge drift simulation. A 266 nm pulsed laser illuminated nine aluminium strips (6 mm wide) placed along the inner cathode of the detector. This generates photoelectrons at well-defined *z* and *ϕ* positions and at known times. Extended Data Fig. 5 shows good agreement between the calibration data and the simulation. The calibration also served to monitor variations of drift time influenced by environmental conditions throughout the measurement campaign. The track reconstruction analyses, performed by artificially varying the Garfield++ model values within the range indicated in Extended Data Fig. 5 (top and bottom panels), did not produce any significant changes in the reconstructed vertices, confirming the validity of our understanding of the detector and its robustness against the possible variations in operational conditions.

### Field measurement and modelling, magnetic biases

#### Electron cyclotron resonance

In electron cyclotron resonance (ECR) magnetometry the magnetic field is deduced from the response of a test cloud of electrons to microwave radiation near its cyclotron frequency. The temperature of a single such test cloud, subjected to a single frequency of microwave radiation, is destructively measured through slow extraction to a microchannel plate and phosphor screen assembly^52^. A spectrum can then be mapped out by rapid repetition of such single exposures using the reservoir technique^53,54^ while sweeping the microwave frequency. We fit a Gaussian function to the spectrum to extract the peak frequency. Here, we apply no evaporative cooling to the test clouds before exposing them to microwaves. This serves to minimize the radial extent (around 0.1 mm) of the test clouds and consequently their sensitivity to radial field gradients. This is necessary in the highly inhomogeneous magnetic fields in regions of the trap that are crucial to the current work. The microwave radiation is produced by a Keysight E8257D synthesizer, with a frequency resolution of 0.01 Hz and an amplitude accuracy, in the parameter range of interest, of ±1.3 dB.

#### ECR during the measurement trials

During each of our experimental trials, we measure the magnetic field simultaneously at two fixed locations near the axial centres of the A and G coils immediately before and after the ramp-down of the mirrors, and again after zeroing all the currents in the internal magnets. This last measurement serves to monitor the stability of the background field over the course of many such trials. The simultaneous measurements are achieved by extracting and positioning two test clouds at a time and irradiating both with the same microwave pulse.

The measurements before the mirrors ramp down display a broadened spectrum due to the high field gradient and have a full width at half maximum of order 7 × 10^−5 ^T, while measurements in the final well and background (external solenoid only) magnet configurations have a full width at half maximum of order 2 × 10^−5 ^T. While significantly smaller spectral widths can be achieved by tuning the microwave parameters and the test clouds, the settings used in this work were chosen to encompass many of the current configurations in the same linear frequency sweep and to ensure robustness against small changes in the loaded reservoir across many experimental trials.

#### Rapid cycle ECR measurements

**Extended Data Fig. 6:**
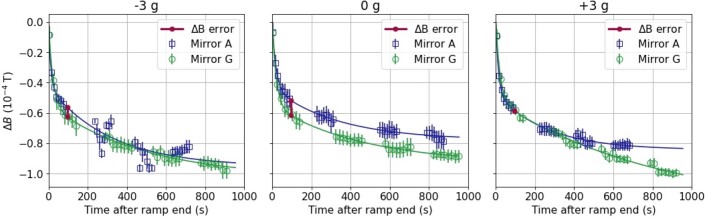
Decay of persistent fields (offline measurements). The on-axis fields at the axial midpoint (Fig. 2a) of mirrors A and G, as measured by the rapid cycle ECR technique (Methods) to study the decay of persistent fields after the end of the 20 s ramp. The solid lines are fits using two exponential decay times per curve; see Methods. The three plots are for the extreme biases ±3 *g* and 0 *g*. The red points represent the extracted systematic error in the magnetic field difference between mirror A and mirror G at each bias, and they are plotted at the approximate time of the ECR resonance in the actual gravity trials. ‘Offline’ refers to measurements taken independently of the release experiments.

The repetition rate of obtaining an ECR spectrum is limited by the time it takes to load and prepare the reservoir from which we extract the test clouds. For the measurements before and after the gravitational release ramps, we extract, expose, and dump 200 clouds to produce simultaneous field measurements near the mirror A/G coil centres in 67 s. By comparison, the reservoir was loaded in around 75 s. A faster repetition rate was obtained by using a reduced set of 25 microwave exposure frequencies to produce eight repeated measurements from the 200 test clouds. In addition, with careful tuning of the reservoir and the test cloud extraction, we can also extract more test clouds from a single reservoir; see the magnetron frequency magnetometry section below. As illustrated in Extended Data Fig. 6, we used this technique to track the decaying field immediately after the end of ramping down the mirror coils. The reservoir was loaded during the magnet ramp, so the resonance was hit within 3 s of the ramp completing. The fits to the data are sums of two exponential decays with differing time constants (roughly 20 s and 300 s).

#### Field measurement using the electron magnetron frequency

We have developed a technique that uses the magnetron frequency of an electron plasma as a measure of the magnetic field at various axial positions in the ALPHA-g device. The measurements described below are taken offline. Using the reservoir technique^53,54^ we extract two thousand reproducible ‘electron clouds’, each containing about 1,000 electrons at a temperature of 100 K and a radius of 100 μm. Although patch potentials (unprogrammed potentials due to, for example, charged oxide layers) and voltage offsets cause the trapping potential to differ from an electrostatic model by about 1%, these potentials are reproducible from day to day to at least one part in 10^5^. When a cloud is radially displaced from the trap centre and trapped by an electrostatic potential *V*_T_ approximated by ${V}_{{\rm{T}}}\left(z,r\right)={k}_{2}({z}^{2}-{r}^{2}/2)$, where *k*_2_ is determined by the electrode potentials, it orbits the centre at a frequency *ω*_r_ , given by ${\omega }_{r}={k}_{2}/| B|$. Precise measurements of this frequency are performed in the following way:

1. A cloud is extracted from the reservoir and moved axially to the desired measurement location.
2. Patch potentials introduce a transverse electric field in the otherwise cylindrically symmetric Penning trap. When the trapping potential is weak (*O*[0.5 V]), the magnetron orbit is no longer centred^55,56^. We quickly decrease the trapping voltage and wait (about 10 ms) for the cloud to arrive at a desired off-axis location.
3. The trapping voltage is then quickly increased, and the cloud begins to orbit the trap centre. After a variable amount of time, it is released towards one of the multichannel plate (MCP) detectors (Fig. 1). The final magnetron phase can be extracted from the cloud’s imaged position.

A single image does not suffice as a measurement of the magnetron frequency because the cloud’s total number of orbits is ambiguous. First, we image one cloud that orbits the trap for a short time *T*_0 _≈ 100 μs. Then we image *N* clouds that orbit in the 1 T magnetic field for a time ${T}_{i}={T}_{0}+{1.4}^{n}\pi (1\,{\rm{T}})/{k}_{2}$, for ${n}=1$ to *N*, that is, geometrically increasing the hold time. For several reasons, there is a variability in the final angular position of about 0.1–0.4 radians depending on the axial location of the measurement. The constant 1.4 is chosen such that before each measurement, the estimate of ${\omega }_{r}$ is good enough that there will be no ambiguity in how many times the cloud orbited the trap centre. In this way, we can increase the total magnetron phase angle while having a roughly constant error. To extract the magnetic field from a precise measurement of ${\omega }_{r}$, we calibrate ${\omega }_{r}$ at a particular field measured with ECR, and we use the relationship ${\omega }_{r}\propto 1/| B|$ to measure the field in the presence of different magnet currents. Of course, there are corrections to this relationship, which are at most about one part in 10^4^.

This technique has been useful for measuring the magnetic field while the magnet currents are ramping. To do this, we image successive clouds after a time *T*_f _= 2,000(1 T)/*k*_2_ (that is, an amount of time such that the cloud would orbit about 2,000 radians in a 1 T field). As the field decreases, the magnetron frequency increases. Depending on the location in the trap, we perform measurements once every 30–50 ms, which means that, in a 20 s ramp, each cloud orbits the trap at most 5 radians more than the previous cloud. We track the total magnetron angle by initially employing a ‘geometrical increase’ operation before the field changes, then we add the angle deviation between successive clouds.

**Extended Data Fig. 7:**
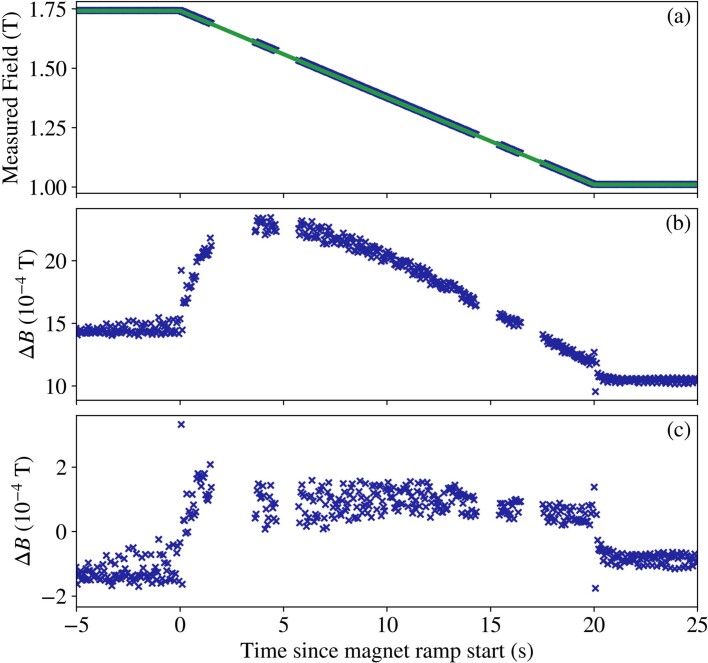
Magnetic field measurements via the magnetron frequency. Magnetic field measured using the magnetron frequency of electron clouds in the centre of mirror A versus time during the magnet ramp-down. **a** Raw measurements (blue) are compared to the expected linear ramp (green line). **b** The difference between the measurements and the expected linear ramp is plotted versus time. **c** Measurements after accounting for the three corrections described in Methods. The 1–2 s gaps in the data are due to a memory limitation in the FPGA that controls the electrode voltages. New voltage instructions are loaded in this time.

Extended Data Fig. 7 shows an example measurement of a 20 s magnet ramp in the centre of mirror A. The first subplot shows the raw measurements. The second shows the result of subtracting the expected model for the magnetic field, which assumes it changes linearly between ECR-measured magnitudes before and after the ramp. The most striking feature is a nonlinear component of about 1 × 10^−3 ^T, which we interpret as persistent currents being induced into superconducting material. When a magnet’s current is decreased by $\Delta I$ from a starting value ${I}_{0}$, we observe a nonlinear component of the field that exponentially saturates with increasing $\Delta I$. For the mirror coils ramp-down used to measure gravity, the field is well approximated by

$$\left|B\right|\left(z,{I}_{0}-\Delta I\right)=A\left(z\right)\left[1-\exp \left(-\frac{\Delta I}{0.1346{I}_{0}}\right)\right].$$

By performing this magnetron magnetic field measurement during 130 s magnet ramps in 20 axial locations, we measured *A*(*z*), and this behaviour of the magnetic field was added to antihydrogen simulations (see below). The *A*(*z*) produced by a mirror coil ramp-down looks similar to the nominal field produced by the mirror coils; it has two bumps centred on each coil. In other words, persistent currents resist a change in the magnetic field. We measure a small difference in *A*(*z*) at the locations of mirror coils A and G that gives rise to the approximately exponentially saturating behaviour of the bias at early times in Extended Data Fig. 6 (see below).

The final subplot of Extended Data Fig. 7 includes three corrections:

1. The exponentially saturating persistent field used in the magnetic field model is subtracted.
2. To image off-centre clouds on the MCP, additional normal conducting magnets near the MCP need to be energized. The 0.6–1.0 mT effect of these magnets is subtracted.
3. The frequency ${{\rm{\omega }}}_{r}$ depends on the distance a cloud is displaced from the trap centre—in part because |*B*| increases off-axis. The correction (about five parts in 10^3^) from this effect is obtained from separate calibration measurements. It takes the form mΔ*I* + b, so the final subplot includes constant, exponentially saturating and linear corrections.

Despite these corrections, the field shows some deviation from the ‘expectation’. First, the deviation is about −0.1 mT before and after the field starts changing. The most likely explanations are errors in the measurement technique that are linear in $$| B| $$ (including calibration error). While the field is changing, there is a positive deviation of 0.1 mT. This is a known effect from the induced current in a nearby magnet. Next, there are exceptional measurement points just after the magnet ramp starts and just after it ends; these are known effects of the magnet control system. There is also a small increase in the first second because the persistent current is not perfectly modelled by an exponentially saturating function. Only this last effect is not included in simulations of the experiment, but it occurs in the same way in both mirror coils and so does not affect the bias. In the end, the magnetron technique provides certainty that there are no other unmodelled effects in the on-axis magnetic field larger than 0.1 mT.

Similar data were taken for several biases at five locations near the centre of each mirror coil. Additionally, the magnetron technique was used to measure magnetic fields in 20 axial locations throughout the trap during the 130 s magnet ramp-downs. These data were useful for identifying and quantifying the exponential saturation of persistent currents. The longer measurement time allowed for a more precise measurement of *A(z)*, which we later verified was consistent with what we observe in 20 s ramp-downs. An upcoming publication will provide a more detailed analysis of these data and description of the measurement.

#### Bias uncertainties

Table 2 lists the estimated uncertainties in our calculation of the on-axis bias. Here we detail how each of those contributions is estimated. Firstly, each ECR spectrum taken exhibits a finite width constituting an uncertainty in the determination of the magnetic field from that spectrum^53^. Since the magnetic field difference (*B*_G_ − *B*_A_) is what enters the bias, we add in quadrature the fitted Gaussian widths from measurements in mirrors A and G. We then average over all valid ECR measurements at the beginning and end of the release ramp to get the ‘ECR spectrum width’ contribution.

The ‘repeatability of (*B*_G_ *−* *B*_A_)’ contribution describes how well the magnetic field difference is repeated from one experimental trial to the next and is evaluated as the standard deviation of all valid bias measurements around the average in each set.

Due to background field gradients caused mainly by the octupole windings, the on-axis field maxima at the end of the ramp are shifted away from the geometric centres of the mirror coils as the currents decrease. We correct for this by mapping out the field maxima with high spatial resolution for every current configuration used (Fig. 2a). Parabolic fits are then used to extract the true locations of the on-axis field maxima (saddle points in 3D), as well as the difference between the field measured at the two fixed locations during the gravity experiments and the true maxima. We take the average absolute residuals of the parabolic fits as an error in this correction, adding in quadrature the errors evaluated in the two mirrors and averaging over all current configurations. This is tabulated as ‘peak field size and *z*-location fit’.

The ‘field decay asymmetry (A to G) after ramp’ uncertainty arises because there is a delay (about 96 s) between the end of the mirror ramp and the measurement of the magnetic field. We expect a slight change in magnetic field in this time due to the decay of persistent currents induced by ramping the magnets. If this decay is not equal in the two mirror coils, there would be an error in the field difference measured. The fast repeat ECR described above allowed us to quantify the field decay and look for any asymmetry in a dedicated measurement that is shown in Extended Data Fig. 6. Here we shift the data to overlap the fitted fields at 0 s and to best highlight any difference in decay rate. We observe a 6 × 10^−5 ^T field change during the first 96 s after stopping the ramp, with no appreciable asymmetry between the two mirrors, nor a strong dependence on the exact current configuration. We take as a potential error the largest observed decay difference between the mirrors out of the three biases investigated.

**Extended Data Fig. 8:**
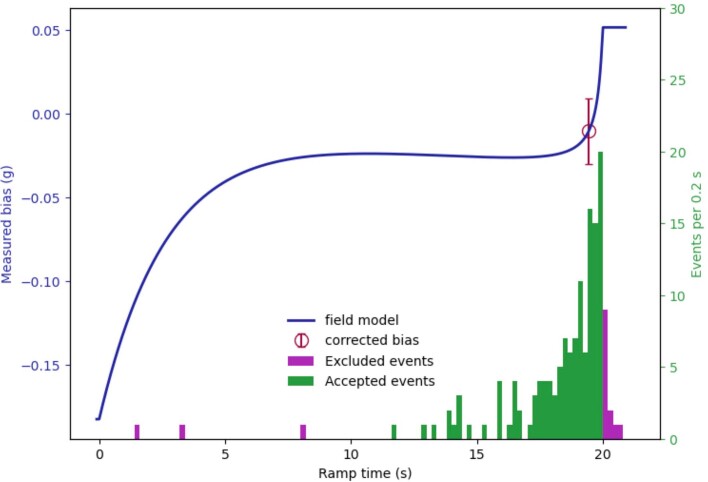
Time dependence of the bias. The solid curve represents the modelled deviation of the bias from the nominal value (in this case *0* *g*) as a function of time during the mirror ramp-down. The histogram shows the number of events detected as a function of time for the *0* *g* trials, as in Fig. 4. The red point shows the derived bias and its uncertainty. Note that the bias deviation during the time data are collected is less than 0.1 *g*. See Methods.

In the main text, we describe how the time-averaged bias for each current configuration is calculated by averaging the calculated bias present in the trap at the time of each annihilation event. This is illustrated in more detail in Extended Data Fig. 8, for a nominal bias of 0*g*. The uncertainty we associate with this spread of biases is the standard deviation of the individual calculated biases of annihilation events. The number given for the ‘bias variation in time’ uncertainty in Table 2 is averaged over all current configurations; individually, they range from 0.010*g* to 0.035*g*.

The bias calculations above rely on a field model to extract the bias at any time during the ramp. The field model is constrained both by ECR measurements of the field at various currents as well as magnetron frequency measurements (see below). To evaluate the accuracy of the on-axis bias in the model, we compared it to offline (that is, independent of the experimental gravity trials) ECR measurements taken in both mirrors at 10 points along the nominal magnet ramps, making sure to match the magnet ramp history and resulting induced persistent currents to the gravity trials. We repeated these measurements for five different current configurations and define the global average of absolute residuals to be the ‘field modelling’ uncertainty.

### Simulations of the dynamics of trapped antihydrogen

#### Field model

A field model was developed to include all knowledge of the magnetic trap during the mirror A/G ramp-down. The model was used to derive the on-axis trap biases and to simulate the three-dimensional trajectories of atoms in the trap.

For the external (1 T) solenoid, an ideal field was first calculated from the designed winding geometry. This was compared to field measurements made with a rubber sample NMR probe in the empty solenoid bore. The difference between the two was deconvolved, using singular value decomposition, to yield current density perturbations on the solenoidal windings. The subsequent installation of the inner cryostat and coils into the external solenoid perturbed its field. The change, mapped on-axis by ECR, was deconvolved into a model solenoidal current distribution overlapping the inner superconducting windings. The ECR-measured background field was replicated in the field model to within 5 × 10^−5 ^T. In the simulation, this background field was assumed to be static during the A/G ramp-down.

The field contributions from the octupoles and mirror coils were computed from winding geometries measured during fabrication. The model windings were slightly offset and scaled to best match the ECR mapping of individual magnets. The currents used in the field model during the A/G ramp-down were measured experimentally using direct-current current-transformers (DCCTs). The experimental current histories had a sample rate of 10 kHz and were filtered by removing Fourier components above 1 kHz before being applied in the field model.

Field measurements made during the mirror A/G ramp, with all windings energized together, revealed field contributions that did not originate from the applied current in any individual winding. We model these contributions in two parts: an exponentially saturating component derived from the magnetron measurements described above, and a residual linear component that further improves the agreement with the aggregate field measurements. These contributions, approximately 10^−3 ^T in magnitude, were included in the field model using a time- and *z*-dependent solenoidal current distribution located approximately where the inner superconducting windings are located.

Putting all contributions together, the field model produced fields that agreed with online ECR, offline ECR and magnetron measurements to a standard deviation of around 2 × 10^−5 ^T overall and around 1 × 10^−6 ^T near the trap saddle points at coils A and G. The former value, converted to units of bias, is quoted in Table 2 as the ‘field modelling’ uncertainty.

#### Trajectory computation

The field magnitudes were precomputed, stored in a regular grid of 0.5 mm spacing, and interpolated via a third-order polynomial for the trajectory simulation. The field interpolation was fractionally accurate to 10^−5^ near the cylindrical vacuum wall where the fields had high spatial variations, and was substantially better away from the wall.

Atoms were evolved in time using a leapfrog stepping algorithm. The time step was chosen individually for each atom and was either 1 μs or an interval such that length traversed per step was no longer than 0.03 mm at all times, whichever was smaller. Stepping was terminated when a trajectory reached the inner Penning trap electrode surface, the UHV beam pipe, or two artificial axial stops located outside the region where atom annihilations are registered by the detector.

The trajectory simulation was made in two parts. (1) To model the initial catching and accumulation process, atoms were initialized near the bottom of the trap. The positions were uniformly distributed over a cylinder of 1 mm radius and 5 mm length. The velocity was drawn from a 50 K Maxwellian distribution. The atoms were initialized with a principal quantum number of 30 and allowed to radiatively cascade down to the ground state using the method described by Topçu and Robicheaux^57^. Each atom was evolved for a randomly selected duration between 0 and 14,400 s to simulate the gradual accumulation of antihydrogen during ‘stacking’. The 6,726 atoms that remained trapped after their specified duration were retained. (2) These atoms were evolved in time through the long octupole and the A/G mirror coils ramp-down using various trap biases and under various assumed gravitational accelerations. The time and location of annihilation were recorded, from which the escape bias curves in Fig. 5, Extended Data Fig. 1 and Extended Data Fig. 2 were derived.

#### Systematic uncertainties

In addition to the escape curves, other results from the simulation have been compared to the experiment. The escape time and axial position distributions of annihilation vertices during the LOc and mirror A/G ramp-down windows showed good agreement. On the other hand, the behaviour of atoms that remained after the A/G ramp-down differed. (Note that these atoms do not contribute to escape curves.) In the simulation, one annihilation in the LOc window corresponded to 0.08 annihilations during the hold after the A/G ramp-down, and 0.51 during the subsequent OcB ramp-down. In the experiment, these numbers were 0.27 and 0.10. This meant fewer atoms than expected survived the A/G ramp-down, and more atoms were driven out of the trap during the hold despite the trap field remaining nominally unchanged.

Given these differences, parameters in the simulation were perturbed to establish the robustness of the escape curve, and to obtain the uncertainty shown in Fig. 5 and quoted in the measured value of the antihydrogen acceleration towards the Earth. We considered the following:

1. The disagreement in the fraction of atoms surviving the A/G ramp-down was found to be consistent with the simulation not having initialized the atoms’ energy in the same way as the experiment. As in our previous work^21^, uniform and linear initial energy distributions were simulated by bootstrapping the results of the nominal 50 K Maxwellian initial energy simulation. The escape curves resulting from these distributions tended to have lower central slopes compared to the nominal curve, but the point of balanced escape remained unchanged. The uncertainty in the simulated escape curve due to this analysis of the total initial energy distribution is included in the uncertainty band in Fig. 5. This demonstrated that the escape curve was not sensitive to even drastic changes to the initial condition of the atoms.
2. The higher-than-expected annihilation count during the hold after the A/G ramp-down was consistent with an energy exchange between the transverse and parallel degrees of freedom that was not predicted. An artificial, unphysical exchange mechanism was therefore introduced to the simulation where atoms received random velocity deflections during their evolution. The strength of this artificial deflection was constrained by the timing of escapes, as excessive exchange forced atoms to escape early. Within this constraint, no changes to the escape curve were observed.
3. Multipolar perturbations with zero component on axis can alter the escape balance of the experiment while eluding ECR and magnetron measurements. Dipole, quadrupole, sextupole and octupole field perturbations were applied to the bottom half (*z* < 0) of the trap to maximize the induced asymmetry. Assuming these perturbations arose from error in the radial positioning of the OcB conductor, the multipolar fields were constrained by the accuracy with which the winding was fabricated (around 10 μm). Assuming the field perturbation arose from persistence effect, the multipolar fields were constrained by the critical current of NbTi. The former resulted in a stronger perturbation and was simulated. The octupole mode perturbation had the most significant impact on the escape curve and effected a maximum $\pm 0.26g$ offset along the bias axis. The central slope was unchanged by the perturbations. The uncertainty (one standard deviation of an assumed flat distribution, Table 3) in the simulated escape curve due to the octupole mode perturbation is included in the orange uncertainty band in Fig. 5.
4. Other field perturbations that were consistent with on-axis magnetometry measurements included transverse offset of the axis of the A and G coils from the OcB axis, and angular misalignment of the external solenoid. These resulted in no change to the escape curve within the mechanical constraints.
5. Mechanical vibration of the trap magnets could heat the trapped atoms and alter their dynamics. This was simulated and no changes to the escape curve were observed at vibration amplitudes below obviously audible/tactile limits.

For each bias value on the escape curve, the largest positive and negative deviations from the unperturbed *P*_dn_ resulting from the above perturbations were chosen for the band displayed in Fig. 5.

#### Magnets and magnet controls

**Extended Data Fig. 9:**
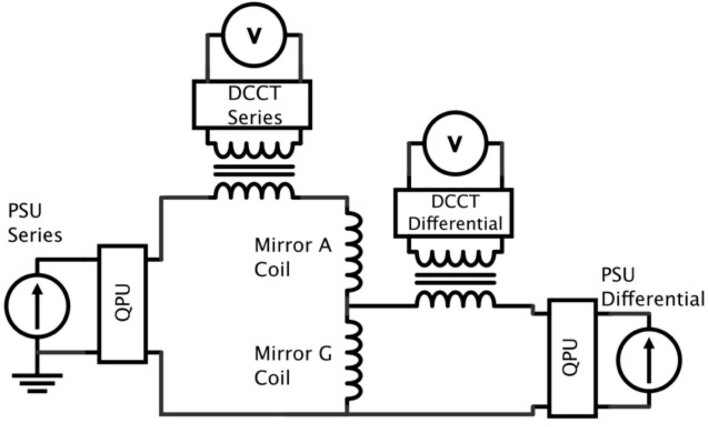
Schematic diagram of the circuit for energizing mirrors A and G (MAG) in series and supplying the differential current (MGDiff) to mirror G only. The power supplies are described in Methods and Extended Data Table 1. QPU stands for ‘quench protection unit’.

The ALPHA-g magnetic trap is generated from superconducting windings housed in two cryostats: the outer cryostat houses a solenoid and shim coils that provide the uniform axial background magnetic field of 1 T needed for plasma confinement in the Penning trap, while the inner one contains 21 distinct superconducting circuits^58–60^. Figure 1 in the main text shows the subset of magnets in use for this study. Mirrors A and G are used to provide axial confinement to the anti-atoms as well as to control the release and are energized in series up to approximately 70 A by a CAENELS FAST-PS-1K5 operating in voltage controlled current supply mode (16-bit analogue to digital input with analogue bandwidth of 1 kHz). An additional, much smaller, differential current is applied in parallel to mirror G alone, using a Kepco BOP 20-10 in voltage controlled current supply mode (analogue input with 10 kHz bandwidth) (Extended Data Fig. 9). We label the series and differential circuits as MAG and MGDiff respectively. This connection scheme ensures that any noise or drift in MAG is shared between both coils and thus has a small impact on the up–down balance of the trap. Extended Data Table 1 details the power supply and performance characteristics of the circuits used in the atom trap region.

We use PM Special Measuring Systems TOPACC Zero-Flux DCCTs installed on the magnet current leads to actively monitor the current supplied to the magnets. The MGDiff circuit was measured using 30 turns of its lead through its DCCT head. Calibrated accuracy of the units is about 25 ppm of the DCCT’s full scale (around 2.5 mA-turn), with less than 1 ppm drift expected over the course of this experiment. Full-scale output of the DCCT is transmitted by a ±10 V signal with an output small-signal bandwidth of 500 kHz. The DCCT output voltages were digitized with 24-bit ±10 V National Instruments NI-9239 cRIO ADC modules at a rate of 50 kS s^−1^. Firmware on the NI cRIO FPGA recorded a running average of this signal at a rate of 10 kS s^−1^. This measurement was used for proportional–integral–derivative (PID)-based closed-loop control of the magnet power supplies (excluding the external solenoid supplies) to compensate for non-linearities in the QPU circuits and internal drift of the power supplies. Current programming voltages for power supplies were generated by NI-9264 analogue output modules with 16-bit resolution. Parallel readout of all monitored and control voltages was recorded at 10 kS s^−1^ by the firmware, with jitter on the order of 1 μs and clock drift relative to the main data acquisition system at the 10 ppm level.

Currents measured during 20 s and 130 s ramp-downs achieved run-to-run repeatability within the operating noise level of the magnet systems (Extended Data Table 1). Deviations from the requested current included a consistent and constant current offset of 1.5 mA during the 20 s linear ramps and 0.22 mA for 130 s linear ramps. These offsets were due to lag in the PID control loop. In addition, a consistent overshoot transient at the start and end of the ramps was produced by the PID control of the MAG series circuit. The deviations of the MAG series current from the programmed linear ramp directly affect the atom trap depth and also introduce a bias field error due to the roughly 1% construction difference between coils. For the 20 s ramp, this was a swing of less than 80 mA (bias 0.017*g*) over approximately 200 ms at the start of the ramp and less than 65 mA at the end of the ramp (bias 0.014*g*, or 12% of final well depth). For the 130 s ramp the start transient was less than 15 mA (bias 0.0032*g*) over 200 ms and less than 12 mA (bias 0.0025*g*, 2% of final well depth) over approximately 200 ms.

During release measurements, currents were inductively coupled into mirrors B, C, D, E and F (unpowered and shorted through resistors), though not in the Background and Transfer coils (disconnected during this study). The respective currents in mirrors B through F were measured during release ramp-downs to be 7.9 mA, 2.6 mA, 2.1 mA, 2.6 mA and 8.1 mA during 20 s ramps, and 1.2 mA, 0.4 mA, 0.3 mA, 0.4 mA and 1.3 mA during 130 s ramps. These contribute to bias magnetic field errors at a level well below 0.01*g*. All measured currents were included in the numerical simulations of the experiment.

#### Analysis for escape curve and gravitational acceleration

The analysis begins by aggregating the time and axial location of antihydrogen annihilations reconstructed during the mirror ramp-down for each bias. Next we apply the *z* and time cuts, described in the main text, to the data. Using experimental calibration samples with biases of −10*g* and +10*g*, for which antihydrogen is largely forced to escape upwards or downwards, we calibrate the efficiencies in the up and down regions of the detector. The cosmic background rates across the trap are constrained using data obtained while the trap is empty.

We perform a likelihood analysis^61^ to determine the probability to escape downwards, *P*_dn_ (or equivalently the asymmetry *A* between the downward and upward escaping anti-atoms *A* = 2*P*_dn _− 1), at each bias. The credible intervals for *P*_dn_ are shown in Fig. 5.

Using the simulation, we then find the set of simulated downward escape probabilities, *P*_sim_, at the measurement biases, for a range of simulated values of the gravitational acceleration *a*_gsim_, and perform a likelihood analysis on the experimental data to estimate ${a}_{\bar{g}}$. The results are cross-checked by repeating the analysis with different fiducial cuts in *t* and *z* and with the 130 s ramp data.

We estimate the significance of having observed the effect of gravity on antihydrogen from the asymptotic distribution of the likelihood ratio between the models with zero and the extracted value of ${a}_{\bar{g}}$.

Counting statistics are included in the likelihood analysis by assuming that the counts in the mirror release in the up and down regions and the LOc counts at each bias are sampled from independent Poisson distributions with the mean specified in terms of the experimental parameters.

Systematic uncertainties are included by allowing the parameters that enter the likelihood analysis to vary according to their experimental uncertainties (where available) or within plausible ranges. The dominant source of systematic uncertainty in estimating *P*_dn_ is the calibration of the detector efficiencies in the up and down regions. The dominant source of error in calculating ${a}_{\bar{g}}$ is related to errors in the simulation model arising from uncertainties in the off-axis magnetic field. Table 3 provides a breakdown of the contributions considered for the total uncertainty.

## Online content

Any methods, additional references, Nature Portfolio reporting summaries, source data, extended data, supplementary information, acknowledgements, peer review information; details of author contributions and competing interests; and statements of data and code availability are available at 10.1038/s41586-023-06527-1.

## Figures and Tables

**Extended Data Table 1:**
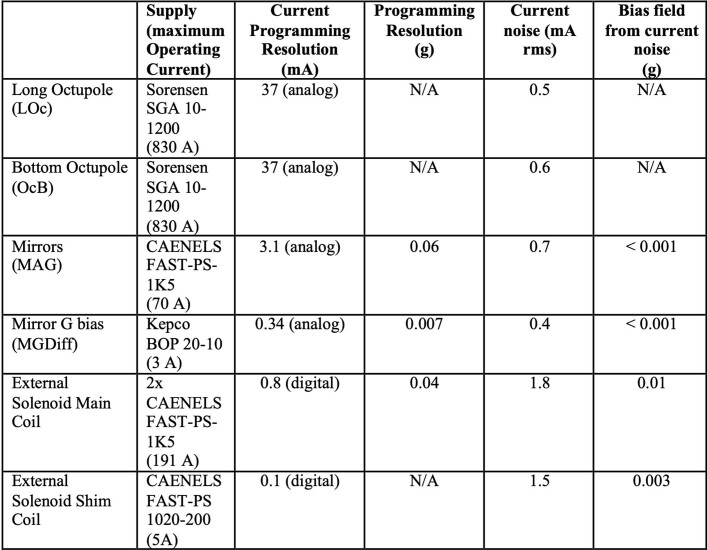
ALPHA-g magnet power supplies

The power supplies used for energizing ALPHA-g magnets are tabulated, together with some of their relevant performance characteristics. Resolution and noise values are expressed in units of the local acceleration of gravity for matter (9.81 ms^−2^).

## Data Availability

The datasets generated during and/or analysed during the current study are available from the corresponding author (jeffrey.hangst@cern.ch) on reasonable request.

## Code availability

The codes used for modelling or analysis in the current study are available from the corresponding author (jeffrey.hangst@cern.ch) on reasonable request.
